# Employment protection and regional self-employment rates in an economic downturn: a multilevel analysis

**DOI:** 10.1007/s00168-023-01214-5

**Published:** 2023-02-25

**Authors:** Nikolaos Filippopoulos, Georgios Fotopoulos

**Affiliations:** Department of Economics, University of Peloponnese, 22 100 Tripolis, Greece

**Keywords:** L26, K31, R10, R23, C19

## Abstract

This research aims to investigate the role of employment protection in affecting the relationship between regional self-employment and unemployment during turbulent times. In doing so, data comprised of 230 regions, nested in 17 EU countries, for the 2008–2015 period were used. When accounting for individual effects, we find that an increase in regional unemployment would decrease regional self-employment, while the opposite was found true for employment protection. When accounting for the cross-level interaction between regional unemployment and national employment protection legislation, however, we find that the underlying increased labor market rigidity not only decreases regional self-employment, but it also magnifies the adverse effect of regional unemployment. Our key results thus indicate that high labor market rigidity hinders self-employment.

**Supplementary Information:**

The online version contains supplementary material available at 10.1007/s00168-023-01214-5.

## Introduction

The aim of this research is to investigate the role of employment protection in affecting the movements of regional self-employment rates during a period characterized by high unemployment spells. In particular, and although we control for a number of labor market institutions, we focus on the role of employment protection legislation in affecting the relationship between regional self-employment and unemployment. In this respect, we consider whether the degree of employment protection can explain the movements of regional self-employment better than regional unemployment, or if there is a joint effect.

The motivation of our research is twofold. First, we aim to add new insight to the literature on the impact of employment protection on self-employment, by turning our attention on its regional implications. Generally, while stricter employment protection can both decrease incentives for self-employment (e.g., Kanniainen and Vesala 2005) and lead to dependent self-employment (e.g., Parker 2010), its effect remains ambiguous (see Golpe et al. 2008), especially given the deregulation of the European labor markets over the recent years (see Heyes 2013; Golpe et al. 2008). Moreover, it could be the case that some labor market institutions (like employment protection legislation) may have played an essential and unprecedented role in determining self-employment during the recent recession, while it is also fruitful to examine the relevant regional implications (Román et al. 2011a). Other studies have also stretched that the effect of labor market institutions is less examined under financial shocks and that it can vary subject to the business cycle (see Boeri and Jimeno 2016; Duval et al. 2020; Ferreiro and Gomez 2020). Thus, and given those considerations, in this study we focus on a unique recessionary period (i.e., the Great Recession).

Second, the research draws on two important suggestions that stress that entrepreneurship, within or across countries, should be examined in relation to the economic and institutional environment (Acs et al. 2008), and that national regulations affect entrepreneurs nationwide who must comply (see Audretsch et al. 2019 for a relevant analysis at the country and city levels). Although in a different context, other regional studies also emphasize on the fact that national laws and institutions affect all regions (e.g., Di Vita 2018; Geppert and Stephan 2008). Other authors have highlighted the fact that the broad macro, regulatory and institutional environment has an effect on entrepreneurial activity which may not be uniform across regions (see Bosma and Schutjens 2011; Fritsch and Storey 2014), which could be due to certain endogenous characteristics (e.g., human capital, industry composition) differentiating regions across and within countries and their ability to adapt to changes brought about by national institutions and conditions. We thus follow and apply these suggestions to the context of our study, by primarily examining the ability of employment protection legislation, which is a national regulation, to influence the relationship between regional unemployment and self-employment. Increased unemployment and strict employment protection signal increased labor market rigidity, which can on its own affect self-employment (Barbieri 2001).

To address the above-mentioned considerations, we use a multilevel analysis. In doing so, we utilize data from 230 regions of 17 EU countries, for the period 2008–2015, and investigate the ability of increased employment protection to affect the relationship between regional unemployment and regional self-employment, during an economic downturn. In addition, we also control for a number of variables both at the regional and country level, put extra weight on the latter level however, where we focus on institutional variables.

Our key results indicate that the direct effect of an increase in regional unemployment would be a decrease in regional self-employment, while the incidence of increased employment protection magnifies the overall negative effect of unemployment. In terms of the direct effect of employment protection, our results indicate that stringent labor legislation pushes into self-employment. We also find, however, that the effect of employment protection is not independent of the level of unemployment either. Specifically, we show that once a certain level of unemployment has been reached, the positive direct effect of increased employment protection is reversed. Other important results highlight the opportunity cost of entrepreneurship during the recent recession and provide evidence that consensus among social partners regarding wage bargaining assists the occupational choice of self-employment. Finally, greater access to finance is positively related to self-employment.

Moving forward, this paper offers the essential conceptual framework which forms its basis (Sect. 2). Next, the data and methods employed to answer the research questions are presented (Sect. 3) before the results are described in detail (Sect. 4). Finally, the paper closes with a round-up of the conclusions and assumptions drawn from the present effort.

## Theoretical background

### Unemployment and self-employment

Entrepreneurship occurs primarily at the local/regional level, since actors interact with local conditions that form the regional entrepreneurial environment, which can be diverse from the entrepreneurial environment of other regions (e.g., see Barreneche García 2014; Bosma and Schutjens 2011; Feldman 2001; Fritsch and Storey 2014; Georgellis and Wall 2000; Konon et al. 2018; Saridakis et al. 2020). For instance, and as Georgellis and Wall (2000) explain, regions differ in their suitability for entrepreneurship (relative to paid employment) which is attributed to factors like industry composition. Other factors, like the local human capital or the market potential, are also responsible for differences among regions (see also Wyrwich 2014). Moreover, notice that different regions exhibit different levels of self-employment persistence, as for example has been reported by Fotopoulos and Storey (2017) for the case of England and Wales, and by Fritsch and Wyrwich (2014) for the case of Germany, shifting the analysis from the national level to the regional one.

Among other factors, such as the stock of human capital, the regional wealth, and industry composition, an important determinant of regional entrepreneurship is the level of regional unemployment (see Müller 2016). Research on the relationship between unemployment and entrepreneurship, however, has always been a complex task to handle, since there are strong theoretical and empirical arguments supporting both the ‘recession-push’ and the ‘prosperity-pull’ effects (see Audretsch et al. 2015; Storey 1991). The ‘recession-push’ effect relies mostly on poor aggregate conditions and increased unemployment spells that impede individuals from obtaining paid employment, thus forcing them to consider self-employment instead. Other factors, such as the access to cheap labor (see Konon et al. 2018) or equipment (see Parker 2018, p. 267) during a recession, can also encourage self-employment. On the other hand, the ‘prosperity-pull’ effect argues for the expected returns from entrepreneurship. Although during times of economic growth employment prospects are better and higher wages increase the opportunity cost of self-employment, it can also be expected that in a booming economy individuals can be pulled by opportunities due to higher product demand and thus consider self-employment. On the contrary, under an economic regime of falling incomes and rising unemployment rates, transitions to self-employment are less probable to occur, since, generally, during economic downturns, firms, regardless of their size, tend to face lower demand for their products. Thus, increased unemployment can signal less favorable market conditions and therefore discourage entrepreneurship (see Fotopoulos 2014).

In terms of the effect of regional unemployment on regional self-employment, evidence is mixed. For instance, in an earlier study for UK regions, Georgellis and Wall (2000) have found both push and pull factors that were subject to the level of unemployment in each region. As such, the authors argue for nonlinear effects. Similarly, and by examining business formation, Hamilton (1989) also argues for the nonlinear effect of unemployment (see also Storey 1991). Robson (1998), on the other hand, did not provide evidence in favor of the ‘recession-push’ effect in UK regions. Cueto et al. (2015), using Spanish data, found that self-employment decreases in a region when the unemployment rate increases, but increases when the unemployment rate in neighboring regions increases. Golpe and van Stel (2008) found a positive effect of unemployment on self-employment for higher-income regions in Spain, while no similar effect was found in lower-income regions.

In a different context, Bosma and Schutjens (2011) examined entrepreneurial activity and entrepreneurial attitude in 127 regions of 17 European countries during 2001–2006 and found that increased unemployment negatively affects perceived business opportunities, and, as their regression results suggest, to some extent established business ownership (firms operating more than 3.5 years) as well. Finally, an important contribution to the relevant literature, while at the country level, is that by Parker and Robson (2004). The authors examined a sample of 12 OECD countries between 1972 and 1996 and found no evidence for a significant interaction term between unemployment and the replacement rate (unemployment benefits). Their contribution, however, lies on the underlying potential mechanism implied by the unemployment rate and an institutional variable, the latter thought to be able to mediate the effect of unemployment on self-employment.

Notice however that the above-mentioned theoretical and empirical considerations offer a general explanation of the possible effect of unemployment during boom-and-bust periods, whereas it does not account for their magnitude, which could be extremely useful when examining severe recessionary periods. That essentially means that our understanding of how unemployment might push into self-employment depends heavily on the period under examination (e.g., see Congregado et al. 2012).

Fritsch et al. (2015) identify such a mechanism when reporting for Germany (during 1996–2010) that although the relationship between unemployment and entrepreneurial entry rates is counter-cyclical, no significant (push) effect is found under unemployment above the trend. Konon et al. (2018) provide similar evidence for German regions for the period 1996–2008, but they also notice that it would be important to consider the effect of more severe economic downturns, such as the one following 2008. Therefore, even if both studies examine the case of Germany which performed relatively well during the Great Recession, they also refer directly to diverse implications under profound economic hardships.

Those implications could be more evident in labor markets that were vigorously hit during the Great Recession. For instance, Novejarque Civera et al. (2020) used data for Spanish regions and found a consistent negative effect of unemployment on firm creation rate, before and after 2008, as well as after controlling for the crisis at the national level. Another example is USA, where there has also been considerable effort to understand how the Great Recession affected entrepreneurial activity (e.g., Fairlie 2013; Fossen 2020). Generally, and as regards the trend in the self-employment rate during 2008–2015, it can be argued that there was a decrease in self-employed with entrepreneurial incentives (see Fondeville et al. 2015; Eurofound 2017), but it must also be noticed that there were heterogeneous trends in the EU member-states. Namely, there were countries that experienced an increase in self-employment (e.g., the Netherlands, UK), others a decrease (e.g., Portugal), while some others only marginal changes (e.g., Austria, Denmark) (see Eurofound 2017). Of course, those heterogeneous trends are also evident within each country, since there are regions diverging from the general patterns pertaining to the country they belong to.

While the inconclusiveness of the relevant literature is obvious, which however depends on factors like the micro- or macro approach, or the labor market under examination, recent studies agree on the unique role played by the magnitude of the Great Recession. Moreover, and as explained by Pissarides (2013), the Great Recession was not a ‘normal’ recession, especially for the European South, which was depicted in the severe rise of unemployment and the sharp fall in aggregate demand.

Thus, considering both the impact of the recent recession on the European labor markets and the suggestions on the role of a severe recession on entrepreneurship (Fritsch et al. 2015; Konon et al. 2018), it seems rational to assume that in Europe while self-employment could have acted as a last resort, the implied lower product demand following the extensive job destruction in that period could not have made entrepreneurship attractive. Moreover, and although the individual incidence of unemployment can be an important determinant pushing into self-employment, the degree of aggregate unemployment of the local labor market cannot be neglected since it can negatively affect self-employment, as the evidence of Berglann et al. (2011) suggests. In addition, the evidence provided by Georgellis and Wall (2000) suggests that in regions with already increased unemployment, additional increases can discourage self-employment and lead to prosperity-pull implications. The above-mentioned considerations indicate that poor aggregate conditions lead to an unfavorable environment for self-employment, especially in regions hit most during the Great Recession (see Novejarque Civera et al. 2020), given the rise of unemployment and the subsequent fall in aggregate demand (Pissarides 2013).

#### Hypothesis 1:

Prosperity-pull implications: An increase in the unemployment rate will negatively affect self-employment.

### Employment protection, unemployment, and self-employment

Apart from the effect of factors such as the regional unemployment rate, or other endogenous factors, scholars often argue that there is a role for the institutional context as well when conducting a regional analysis. For instance, Agostino et al. (2020) explain that there is a trade-off between safe-salaried employment and higher and more volatile returns from entrepreneurial activity, which is affected by the institutional quality. The authors further explain that in a non-crisis context higher institutional quality decreases the risk associated with entrepreneurship, while in times of crisis the already risky choice of entrepreneurship might not be an option even under good institutions. Moreover, and as argued by Bosma et al. (2018), institutions can determine under what conditions entrepreneurs can utilize inputs such as labor, finance, and knowledge to generate output (see also Agostino et al. 2020).

However, and apart from the role of institutions on entrepreneurship in general, we must also examine whether national institutions exert any kind of influence on the regional entrepreneurial activity (see Bosma and Schutjens 2011; Wyrwich 2014). For instance, Wyrwich, (2014) explains that the institutional shock related to the transformation of post-communist regions into market economies had a positive impact on start-up activity. Audretsch et al. (2019), and although focusing on the country and city levels, argue that there are indeed (business) regulations governing all entities, which of course shape entrepreneurship, which occurs locally and comes with certain local constraints. Similarly, Di Vita (2018), and in a different context, argues that while regions have their own economic and institutional individuality, we cannot ignore that they also have to comply with the national laws (again in a different context, see Geppert and Stephan 2008 for accounting for national effects in their regional analysis). Elhorst and Zeilstra (2007) also criticize the fact that some studies focusing on regional-level variables essentially treat regions as independent entities.

Therefore, the institutional implications brought about by national regulations may affect differently regions even within the same country and thus more attention must be paid. In particular, and within the context of the present study we turn our attention on employment protection. Apart from the effect of unemployment, considerable attention has been paid on the role of rigid labor markets, especially given that employment protection is an important determinant of self-employment (Hipp et al. 2015). Of course, the effect of employment protection may be diverse subject to the level of strictness of the relevant legislation, as shown by Román et al. (2013), who examine the probability of entering self-employment from unemployment under the occurrence of additional increases in employment protection (both in flexible and rigid labor markets). In addition, and as with the unemployment rate, evidence on the effect of employment protection legislation is, again, mixed.

Some authors argue that increased employment protection could negatively affect self-employment (e.g., Kanniainen and Vesala 2005). In this sense, stricter regulations in hiring and firing are often considered to be an obstacle for employers in deciding how much labor is needed for firms to operate, and thus discourage entrepreneurship. Moreover, strict hiring and firing regulations can also provide disincentives to potential self-employed who know that, in the case of business failure, it can be hard to return to paid employment (Golpe et al. 2008). Van Stel et al. (2007) explain that, under flexible markets, job insecurity can push employees into entrepreneurship, while employers enjoy this labor market flexibility in order to run their businesses. Thus, and according to the authors, increased regulatory rigidity decreases employer flexibility and increases the costs associated with potential dismissals. Notice that this decreased flexibility, and the increased labor costs associated with it, can lead to less employment given the reluctance of employers to create new jobs (Kahn 2007).

On the other hand, the direct effect of a rigid labor legislation that induces the slanginess of the labor market and impedes job creation, could be also expected to lead to push effects (e.g., see Román et al. 2013). A positive relationship can thus emerge in tighter labor markets where alternative forms of employment (i.e., dependent self-employment) act as a counterweight to strict employment protection legislation (Parker 2010; Román et al. 2011b, 2013; Ulceluse and Kahanec 2018). Moreover, self-employment could act as a last resort. The rationale is that employers cannot easily dismiss the redundant labor, while simultaneously limiting themselves from hiring more, thus pushing individuals who cannot obtain paid employment into self-employment.

Nevertheless, it is also important to consider the existing economic conditions when investigating the effect of labor market flexibility, since a recession and strict employment protection may favor dependent self-employment, or self-employment as a last resort (i.e., employers do not hire labor both due to the recession and the unfavorable legislation). In fact, Román et al. (2011a) argue that the coexistence of a recession and strict employment protection may lead to dependent self-employment, or self-employment as a last resort. In view of the above, and taking into account the magnitude of the Great Recession and the insight provided by Román et al. (2011a), we proceed to the next hypothesis:

#### Hypothesis 2:

Any increase in the strictness of employment protection urges self-employment.

Notice, however, that much less effort has been made by the relevant literature to identify connections between unemployment and employment protection that could interact and jointly explain entrepreneurship, despite the existence of studies that somehow incorporate, or at least mention, both factors in their analyses. For instance, Bosma and Schutjens (2011) include both regional unemployment and employment protection legislation in their analysis; however, as these are probably outside their research scope, no joint effects are examined.

In addition, the existing literature that employs relevant interactions remains scarce and in a different context. Parker and Robson (2004), for example, examine the interaction between the unemployment rate and the replacement rate, which however accounts for the benefits of unemployment and only indirectly, and loosely, accounts for its relevant implications on workers. For the record, the authors find no evidence supporting the subsequent push effect of unemployment. In a different context, Fu et al. (2018) examine whether labor market regulations could affect ex-entrepreneur re-entry into entrepreneurship, while accounting for their current work status (employed or unemployed). The authors find evidence supporting that currently employed ex-entrepreneurs are more likely to re-enter entrepreneurship, than unemployed ones, attributing this finding to the fact that, while employed under strict labor regulations, employed individuals enjoy the legal safety nets and are less financially constrained.

Another shortcoming is that many studies focus on the individual level. Individual-level transitions from unemployment to entrepreneurship, however, can be hindered due to human and financial capital constraints (Parker 2018, p. 268) and thus not directly or solely related to the level of employment protection and/or aggregate conditions. As such, studies like that by Baumann and Brändle (2012) are more informative since they account for the interaction of employment protection and educational attainment. In addition, evidence shows that while unemployment might be positively related to entrepreneurship at the individual level, the relationship can be negative at its aggregate counterpart (Berglann et al. 2011). That is because the latter is an indication of the aggregate prevailing conditions within a region/country’s labor market, which, in conjunction with the strictness of employment protection, manifests the level of labor market rigidity (Barbieri 2001).

Moreover, it is one thing to interpret the effect of unemployment and employment protection independent to each other in an analysis, but quite another to examine whether the joint effect of these two measures can explain self-employment, especially in the context of a recession. Strikingly enough, this is overlooked by the related empirical research, especially since evidence from the Great Recession shows the potential connections existing between unemployment and employment protection. Evidence, for example, shows that the unemployment rate in Germany during the Great Recession was held at lower levels compared to the rest of European economies, but the overall performance of the German labor market was enhanced by (prior) reforms that aimed to stimulate job search, self-employment and deregulate the job market (Ter Weel 2015; Weber 2015; Pissarides 2013). In this sense, Germany’s prior reforms helped mitigate the adverse unemployment effects of the recession, while countries like Greece and Spain trying to implement fiscal and labor market reforms at the same time were caught in a phase characterized by both a sharp decline in aggregate demand and rigid labor institutions, eventually leading to more unemployment (Pissarides 2013). It is thus evident that labor market institutions (and the reforms related to them), are closely related to the diverse response and impact of unemployment of different economies to the recession.

Given that no direct evidence is as yet available that shows any potential link between increased unemployment and stringent labor legislation that could affect self-employment during a recession, the possibility to build hypotheses based on previous findings and/or theoretical arguments is, at best, limited. Nevertheless, the studies that somehow formally consider both factors, do not provide (strong) evidence in favor of a joint push effect of unemployment and employment protection. As such, we first build on Barbieri (2001) who examined whether self-employment in Italy can be explained by the unemployment rate and employment protection, both high at the time, thus signaling labor market rigidity. The author, however, found no evidence that this labor market rigidity could explain self-employment movements. Second, we consider that Román et al. (2013) find evidence that increased national unemployment and more stringent labor legislation in countries with already strict EPL discourage self-employment with employees, but impel own account self-employment. The latter, however, was found to be only weakly statistically significant according to the regression results. In addition, we consider that i) high aggregate regional unemployment signals less promising entrepreneurial prospects (Bosma and Schutjens 2011; Georgellis and Wall 2000; Novejarque Civera et al. 2020), and ii) strict labor regulations lead to increased labor dismissal costs (van Stel et al. 2007), jointly implying an overall labor market rigidity (Barbieri 2001) (i.e., product markets are in a downturn and firing and hiring comes with increased legislative obstacles), and thus, at the end of the day, ‘prosperity-pull’ implications (Audretsch et al. 2015; Storey 1991). We thus proceed to Hypothesis 3:

#### Hypothesis 3:

Any increase in the strictness of employment protection will magnify the overall negative effect of unemployment on self-employment.

## Empirical strategy

### Data

To investigate the effect of the labor market on regional self-employment rates, we employed data for 230 regions (NUTS-2) nested in 17 EU countries (see Appendix, Table 5) between 2008 and 2015. The timeframe serves two purposes. First, it includes both the core recessionary years of the Great Recession and the follow-up recessions triggered in most of the EU Member-States. The Great Recession was not a typical recession as it caused an unprecedented fall in economic activity as well as significant financial restraints for small firms (see Angulo-Guerrero et al. 2017; Haltiwanger 2022; Pissarides 2013). Using the COVID-19 pandemic crisis as an example, and as Haltiwanger (2022) argues, financial markets were in better shape than they were during the Great Recession, and the growth of remote operations made it possible for new businesses to profit from this economic restructuring. This was, most certainly, not the case during the Great Recession when businesses faced severe financial constraints and had no visible means of adjusting to the adverse economic shock. Second, the handling of the Great Recession is closely related to the quality of labor market institutions that had to adjust (or were already able to adjust) to the financial crisis. Different countries encountered different challenges in boosting business and economic activity, in addition to lowering unemployment. As already noted, the adverse effects of the crisis hit differently in countries that were implementing labor market reforms during the crisis and alongside other fiscal reforms, and differently in countries with an already more flexible labor legislation and better aggregate conditions (see Ter Weel 2015; Weber 2015; Pissarides 2013).

Regarding the predictors, and starting from the set of regional variables, the sources are Eurostat and the European Commission (ARDECO), which provide full information on many important indicators. The obvious advantage of both databases is the availability of scarce regional data, making the investigation of cross-country differences regarding the regions of EU countries possible. At the country level, we exploited the information provided by the European Commission, the ICTWSS database version 6.1 (Visser 2019), the OECD and the World Bank.

#### Dependent variable

Using the data provided by Eurostat, our dependent variable is the regional self-employment rate (age group 15–64) (SER), as measured by the regional sum of the self-employed divided by the labor force. Although an imperfect measure, self-employment is widely used in entrepreneurship research due to the availability of data, its ability to conduct international comparisons and, most importantly, due to its ability to account for firm owners and the risk inherent to entrepreneurship (see Georgellis and Wall 2000; Low and Weiler 2012; Parker 2018, p. 16; Saridakis et al. 2014; Ulceluse and Kahanec 2018). Finally, notice that the sample size restrictions put by the method used in this paper did not allow for the utilization of alternative measures (see Sect. 3.2). For these reasons, and given the aggregate nature of our dependent variable, we utilize and treat the self-employment rate as the net occupational choice related to entrepreneurship made within a region.

#### Independent variables at the regional level

The main variable of interest at the regional level is the regional unemployment rate (age group 15–64) (UNEMP) and was extracted from Eurostat. UNEMP is used to account for ‘recession-push’ and ‘prosperity-pull’ dynamics, and the response of employers to negative shocks. However, we also use as control variables the regional GDP per capita (PPS) (GDP p.c.), the industry composition (employment in five sectors; % of total employment; NACE Rev.2; agriculture excluded) (European Commission, ARDECO), the human capital (HC) (age group 25–64; % of population with tertiary education) (Eurostat), the population density (persons/km^2^) (PDEN) (Eurostat; European Commission, ARDECO), and the female share in population (FMS) (age group 15–64; % of total population) (Eurostat). GDP p.c. accounts for regional wealth and operates as a proxy for capital per worker. Theoretically, capital-intensive economies can trigger a decline in returns to entrepreneurship relative to wages (Torrini 2005). The industry composition has been argued to be able to explain (at least theoretically) the variation in self-employment (see Acs et al. 1994; Torrini 2005) and to depict the suitability for entrepreneurs in a given region (Georgellis and Wall 2000). HC controls for the human capital empowered with knowledge that could encourage and develop entrepreneurial skills (Barreneche García 2014). Population density accounts for the local market size, which represents the level of opportunities available to entrepreneurs (Audretsch and Keilbach 2007), and also serves as a proxy for agglomeration economies, where, for instance, the occurrence of economic spillovers could drive self-employment (see Goetz and Rupasingha 2014). Finally, the female share in population is a control variable since evidence at the individual level shows that gender is an important determinant of self-employment choice and demonstrates that males are more likely to become self-employed than females (see Le 1999; Simoes et al. 2016), which is a finding further confirmed at the regional level (Georgellis and Wall 2000).

#### Independent variables at the country level

At the country level the variable of interest is Employment Protection Legislation index (EPL) from the OECD and is used to account for the statutory environment regarding the flexibility/slanginess of the labor market.^1^ In addition, we employ control variables at the country level as well. As regards employment protection indexes, like the EPL, one can ponder about their adequacy to represent the level of labor market flexibility and its impact on self-employment. Essentially, one substantial problem is that employment protection indexes might represent the direction of employment protection policies and not their real effects (i.e., law enforcement, employer compliance, or compliance of smaller firms, etc.), since the enactment of a policy is not always equal to its enforcement (e.g., see Boeri and Ours 2008, pp. 214–215; Golpe et al. 2008; Maleszyk 2016; Myant and Brandhuber 2016).

For that reason, it is essential to control for other institutions as well. Along this line, the existence a binding minimum wage also provides additional useful insight on the degree of employment protection. Regarding minimum wages, and according to Parker (2018, p. 700), wage rigidities (i.e., the absence of a downward pressure on wages) brought about by minimum wage can push workers toward self-employment (see also Blau 1987). In addition, employers may also decide to contract out work to dependent self-employed workers in order to combat increased labor costs (Parker 2010). For instance, vom Berge and Frings (2020), in their regional analysis on high-impact minimum wages in Germany, argue that the increase in self-employment in East Germany could be partially attributed to the incidence of dependent self-employment. However, an increase in their level can induce an increase in labor costs, which explains why some empirical evidence suggests the negative impact of minimum wages (e.g., Coomes et al. 2013; Kwapisz 2019). That could be more evident in countries with generous minimum wage rates (e.g., France) during the Great Recession, or in countries, like Greece, where the economic shock radically increased job destruction. Therefore, and according to the information provided in Section B of ICTWSS database, the dummy variable MW accounts for the existence of minimum wage legislation (= 1, 0 = otherwise), in order to control for the legal, wage-related, obligation of employers, and the legal safety net of employees.

On the assumption that we truly consider wages the opportunity cost of entrepreneurship (e.g., see Amit et al. 1995; Foti and Vivarelli 1994; Knight 1921; Oxenfeldt 1943), we might have to control for the forces influencing their level as well, since settling on the true wage level requires a broad consensus among social partners. Consensus can thus indicate coordination. Thus, regarding the institutional environment in which wage bargaining takes place, we construct two dummies based on the information available in Section B of the ICTWSS database. COORD controls for the existence of an environment of increased coordination among social partners regarding wage setting (= 1, 0 = otherwise). GOVINT accounts for the increased government intervention in wage bargaining that exceeds simple consultation and conflict resolution (= 1, 0 = otherwise).^2^ Moreover, both variables account for the heterogeneity in coordinating wages, that is the opportunity cost of entrepreneurship, which is closely related to the Varieties-of-Capitalism (VoC) literature (see Dilli et al. 2018).^3^

Another labor market institution depicting the level of employment protection is provisions enabling labor contract renegotiations (i.e., opening clauses). Yet, and to the best of our knowledge, there are no studies linking opening clauses to the occupational choice of self-employment (maybe except for Carrasco and Hernanz 2021), let alone during the Great Recession. However, and at least theoretically, opening clauses can be an effective tool for employers during recessions in order to adjust labor costs (i.e., wage cuts that are either linked to a subsequent reduction in working hours or not) and prevent job destruction (e.g., see Brändle and Heinbach 2013), which in turn could provide incentives toward self-employment. On the other hand, employees have more chances into maintaining their jobs during a period characterized primarily by job destruction which favors paid employment. Nevertheless, while the effect of opening clauses can be bidirectional, the magnitude of the Great Recession could have probably created a negative link between the enabling of labor contract renegotiations and self-employment, providing employees the chance to maintain their jobs in an unfavorable period for entrepreneurship. Hence, in this study OCT accounts for agreements that contain crisis-related opening clauses, defined as temporary changes, renegotiation, or suspension of contractual provisions, under defined hardship conditions (= 1, 0 = otherwise) (Section B of ICTWSS database).

Finally, we use DCR (domestic credit provided by banks to the private sector, World Bank) as a control variable for the credit available to the private sector, since greater access to finance can affect entrepreneurial decisions, while lack of finance can be an important constraint for entrepreneurship (Naudé et al. 2008). Full descriptions of variables and summary statistics are provided in Tables 1 and 2, respectively, while the correlation matrix is in Appendix (Table 4).

**Table 1:** Description of variables and sources

Variables	Description	Source
*Regional level*		
SER	Self-employment % (age group 15 − 64; Self-employed/Labor force)	Eurostat, Own calculation
UNEMP	Unemployment % (age group 15 − 64)	Eurostat
GDP p.c	GDP per capita (PPS)	European Commission (ARDECO)
HC	Population with tertiary education (%; age group 25 − 64)	Eurostat
PDEN	Population density (persons/km^2^)	Eurostat ^a^
FMS	Female share (% of total population; age group 15 − 64)	Eurostat
IND	Employment in Industry—excluding Construction (% of total employment)	European Commission (ARDECO), Own calculation
CON	Employment in Construction (% of total employment)	European Commission (ARDECO), Own calculation
WRTAC	Employment in Wholesale, Retail, Transport, Accommodation and Food Services, Information and Communication (% of total employment)	European Commission (ARDECO), Own calculation
FBS	Employment in Financial and Business Services (% of total employment)	European Commission (ARDECO), Own calculation
NMS	Employment in Non-market Services (% of total employment)	European Commission (ARDECO), Own calculation
*Country level*		
EPL	Employment Protection Legislation index	OECD
MW	Existence of minimum wage legislation (1 = Yes, 0 = No)	ICTWSS database ^b^
COORD	Environment of increased coordination among social partners regarding wage setting (1 = Yes, 0 = No)	ICTWSS database ^b^, Own construction based on Section B
GOVINT	Increased government intervention in wage bargaining that exceeds simple consultation and conflict resolution (1 = Yes, 0 = No)	ICTWSS database ^b^, Own construction based on Section B
OCT	Existence of crisis-related, temporary opening clauses in collective agreement [1 = agreements (at any level) contain crisis-related opening clauses, defined as temporary changes, renegotiation, or suspension of contractual provisions, under defined hardship conditions 0 = No]	ICTWSS database ^b^
DCR	Domestic credit to private sector by banks (% of GDP)	World Bank

^a^Additional data whether gathered from the European Commission (ARDECO) to compute missing values

^b^Institutional Characteristics of Trade Unions, Wage Setting, State Intervention, and Social Pacts (ICTWSS) database version 6.1 (Visser 2019)

**Table 2:** Summary statistics

Variable	*N*	Mean	SD	Min	Max
SER	1840	0.13533	0.05696	0.05589	0.41075
UNEMP	1840	0.09284	0.05604	0.01900	0.36300
GDP p.c	1840	26,045.02563	8570.90022	9509.35795	59962.36711
IND	1840	0.16086	0.06884	0.04155	0.37590
CON	1840	0.07061	0.01522	0.02859	0.14829
WRTAC	1840	0.27007	0.04433	0.19238	0.50463
FBS	1840	0.14303	0.05047	0.04463	0.28985
NMS	1840	0.30519	0.05280	0.17658	0.44298
HC	1840	0.26793	0.08515	0.06800	0.55700
PDEN	1840	332.84585	697.26940	3.30000	7408.00000
FSH	1840	0.45634	0.02349	0.33877	0.51322
EPL	1840	4.60714	1.20079	2.14400	6.12800
MW	1840	0.65109	0.47676	0	1
COORD	1840	0.56250	0.49621	0	1
GOVINT	1840	0.31033	0.46275	0	1
OCT	1840	0.34620	0.47589	0	1
DCR	1840	1.05646	0.40489	0.29930	2.01259

### Methodology

Given the hierarchical nature of our dataset (years, regions, countries), a multilevel model (MLM) was used to investigate the relationship between regional self-employment rates and country-level labor market policies and institutions. In particular, a three-level model was estimated, where the years are at the lowest level, i.e., level-one, regions are at level-two, which in turn are nested in their corresponding countries, i.e., level-three. The inclusion of the third level is rather important, since ignoring a higher level in a nested analysis can, for instance, prevent the identification of non-null effects and add the variance component of the ignored level to the component of the new higher level (see McNeish and Wentzel 2017; van den Noortgate et al. 2005; van Landeghem et al. 2005). However, given that multilevel modeling can be very demanding in terms of sample size, especially at the highest level of hierarchy (e.g., see Bryan and Jenkins 2016; Hox et al. 2017, pp. 212–218; McNeish and Wentzel 2017), we do not include random slopes, but we include random intercepts to account for the unobserved heterogeneity in both the regional and country levels.^4^

The MLMs of interest are Eqs. 1 and 2. Starting with the first, we estimate the effect of level-two and level-three variables on regional self-employment rates^5^:

1 $${\mathrm{SER}}_{tij}={\gamma }_{000}+{\sum }_{p}^{n}{\gamma }_{0p0}{X}_{ptij}+{\sum }_{q}^{n}{\gamma }_{00q}{Z}_{qtj} + {u}_{00j}+{r}_{0ij}+{\varepsilon }_{tij}$$

SER_tij_ = self-employment rate at time t in region i in country j.

*X*_ptij_ = level-two predictors at time t in region i in country j.

*Z*_qtj_ = level-three predictors at time t in country j.

*u*_00j_ = residual error at the country level (level-three).

*r*_0ij_ = residual error at the regional level (level-two).

*ε*_tij_ = residual error term (level-one).

The main interest of the analysis is in Eq. 2, however, where we investigate the possible significant cross-level interaction between the regional unemployment rate, UNEMP (level-two), and Employment Protection Legislation, EPL (level-three):

2 $${\mathrm{SER}}_{tij}={\gamma }_{000}+{\sum }_{p}^{n}{\gamma }_{0p0}{X}_{ptij}+{\sum }_{q}^{n}{\gamma }_{00q}{Z}_{qtj} +{UNEMP}_{tij}{EPL}_{tj }+{u}_{00j}+{r}_{0ij}+{\varepsilon }_{tij}$$

All predictors are (grand-mean) centered since centering provides a meaningful interpretation of cross-level interactions (Hox et al. 2017, p. 49). One more substantial methodological parameter is the intraclass correlation *(ICC)*, which accounts for the proportion of variance explained at the country level (Hox et al. 2017, p. 21). A high ICC value indicates that a multilevel approach is necessary. Note that ICC is initially calculated at the first stage of the analysis, where the null model is estimated (no predictors are included). In the context of our three-level model, ICC is:

3 $$\mathrm{ICC}=\frac{{\sigma }_{{u}_{000}}^{2}}{{\sigma }_{{u}_{000}}^{2}+{ \sigma }_{{r}_{000}}^{2}+ {\sigma }_{e}^{2}}$$

where ${\sigma }_{e}^{2}$, ${\sigma }_{{r}_{000},}^{2}$ and ${\sigma }_{{u}_{000}}^{2}$ are the variances at the first, second, and third level, respectively.

## Results and discussion

The results of the multilevel analyses are presented in Table 3. The null model (column 1) has an intraclass correlation coefficient of 0.80358, suggesting a very high correlation of regions within countries. This, in turn, justifies the use of the multilevel analysis. Regarding multicollinearity, no relevant problems occurred. All variables have a VIF < 10 (Kutner et al. 2004).

**Table 3:** Labor market determinants of regional self-employment rates (SER). Multilevel model (MLM) estimates

	(1)	(2)	(3)	(4)
*Regional-level variables*				
UNEMP		− 0.21443*** (0.01199)	− 0.15466*** (0.01363)	− 0.13942*** (0.01426)
GDP p.c		− 0.00074*** (0.00015)	− 0.00070*** (0.00016)	− 0.00070*** (0.00016)
IND		− 0.49999*** (0.03116)	− 0.49522*** (0.03112)	− 0.49849*** (0.03113)
CON		− 0.35955*** (0.04298)	− 0.37476*** (0.04644)	− 0.37965*** (0.04636)
WRTAC		− 0.36198*** (0.03249)	− 0.38098*** (0.03241)	− 0.38376*** (0.03239)
FBS		− 0.40579*** (0.03911)	− 0.37511*** (0.03911)	− 0.36000*** (0.03928)
NMS		− 0.45810*** (0.03397)	− 0.48362*** (0.03401)	− 0.48961*** (0.03403)
HC		0.08595*** (0.01085)	0.09023*** (0.01115)	0.08852*** (0.01113)
PDEN		0.00614*** (0.00180)	0.00458** (0.00183)	0.00390** (0.00184)
FSH		− 0.09628*** (0.03395)	− 0.06577** (0.03328)	− 0.05822* (0.03327)
*Country-level variables*				
EPL			0.00863*** (0.00180)	0.00858*** (0.00179)
MW			− 0.00517*** (0.00150)	− 0.00514*** (0.00150)
COORD			0.01117*** (0.00147)	0.01044*** (0.00148)
GOVINT			− 0.00365*** (0.00113)	− 0.00287** (0.00115)
OCT			− 0.00118* (0.00068)	− 0.00105 (0.00068)
DCR			0.00433** (0.00195)	0.00370* (0.00196)
*Cross-level interaction*				
UNEMP*EPL				− 0.03453*** (0.00991)
Constant	0.13446*** (0.01275)	0.13309*** (0.01085)	0.13215*** (0.01025)	0.13282*** (0.01025)
Country-level variance	0.00270 (0.00097)	0.00197 (0.00071)	0.00175 (0.00064)	0.00175 (0.00064)
Regional-level variance	0.00056 (0.00006)	0.00026 (0.00003)	0.00026 (0.00003)	0.00026 (0.00003)
Residual variance	0.00010 (3.39E − 06)	0.00007 (2.43E − 06)	0.00006 (2.27E − 06)	0.00006 (2.25E − 06)
Average VIF		2.41	2.84	3.00
N	1840	1840	1840	1840
Countries | Regions	17 | 230	17 | 230	17 | 230	17 | 230
ICC	0.80358	0.85868	0.84305	0.84273
Log restricted-likelihood	5418.0408	5731.7208	5753.8059	5756.1522
Wald χ^2^ test		919.55***	1077.56***	1094.82***
LR test vs. linear regression	5523.62***	4142.46***	3830.12***	3826.98***

Standard errors are in parentheses; ***p < .01; **p < .05; *p < .1; a = For presentation purposes PDEN was rescaled to stand for 1000 persons/km^2^, while GDP p.c. was multiplied by 1000

### Push and pull dynamics: Regional unemployment and employment protection

Starting with the direct effects of UNEMP and EPL, in all columns of Table 3, the direct effect of UNEMP supports prosperity-pull implications (Hypothesis 1). Practically, what the direct effect implies is that an increase in unemployment indicates poor aggregate conditions (i.e., low product demand) which can severely discourage self-employment.^6^ On the other hand, a positive association between EPL (that is, an increase in employment protection) and regional self-employment rate is observed (Table 3, columns 3 and 4) (Hypothesis 2).^7^ Given the positive and statistically significant sign, and drawing on the suggestions made by relevant studies (e.g., Parker 2010; Román et al. 2011b, 2013), we assume that under tight markets, i) employers have incentives to contract out work, and ii) the labor force has less chances of obtaining paid employment, thus considering self-employment instead. For this reason, tight labor markets may indeed have the effect of pushing into self-employment.

The cross-level interaction between UNEMP and EPL, however, is negative. The first straightforward implication is that the negative effect of UNEMP is magnified further when taking into account its interaction with the EPL index (Table 3, column 4) (Hypothesis 3). Second, the negative sign of the cross-level interaction term (UNEMP*EPL) indicates both a distorted and a tight labor market (Table 3, column 4). Consider the case where u (the unemployment rate) is above a critical value, say u*, and e (an indicator of employment protection) is above the critical e* as well. When u > u*, poor aggregate conditions indicate low product demand, which can reduce self-employment incentives. When e > e*, it is much riskier for individuals to pursue self-employment, since in this case they would have to face the hostile environment in labor markets too.

Another straightforward implication of the cross-level interaction is drawn from an important insight provided by Golpe et al. (2008), who argue for the incidence of business failure and the role of strict employment protection in deterring transitions back to paid employment once a business has failed. Given that during a recession business failure is highly probable, the onerous environment implied by increased unemployment and strict EPL could act as a disincentive to pursue self-employment amid the fear of business failure. Thus, switching to self-employment (from paid employment), and then once failed trying to return to paid employment, seems a rather risky choice under stringent EPL, the latter hindering job creation (Kahn 2007). On the other hand, the unemployed might choose to continue searching for those scarce jobs during a recession than risking failing (in entrepreneurship) and then returning to unemployment. Both situations lead workers and unemployed persons to “lose their place in the queue” (see Parker 2018, p. 661, for a similar argument) if they close their businesses and try to (re-)search for paid employment. Additionally, the interaction term implies that the financial constraints that the unemployed (or low paid employees) face, hinder transitions to self-employment (i.e., capital is needed to pursue self-employment), while at the same time strict EPL implies that even if self-employment is entered, hiring personnel would come with increased potential dismissal costs.

Thus, and while Barbieri (2001) found no evidence that increased labor market rigidity could push into self-employment, we even find evidence for the adverse combined effect of increased unemployment and EPL. In addition, we also complement the important step made by Román et al. (2013), who take into consideration the implications of unemployment and EPL in a stringent regulatory environment. In turn, our results indicate that the coexistence of adverse conditions in both product and labor markets jointly created disincentives for self-employment during 2008–2015. Of course, the overall negative effect of UNEMP, after EPL is taken into account, differs between economies. The negative joint effect is larger in the regions of countries like France, Greece, Portugal, or Spain, which exhibit high levels of employment protection.

Taking the analysis a step further, the cross-level interaction between UNEMP and EPL implies that employment protection is not independent of the prevailing unemployment rate either. That is, we might have found that EPL affects the overall relationship between unemployment and self-employment, but our results also show that the overall effect of EPL on self-employment is subject to the level of unemployment as well. According to our results, an unemployment rate greater than 24.8% would turn the push effect of EPL (i.e., its positive independent effect) into a negative one. This, in turn, is another indication that an overly hostile environment implied both by increased unemployment and stringent labor legislation would ultimately discourage self-employment. In other words, extreme levels of unemployment diminish the ‘self-employment as a last resort’ possibility implied by the positive independent effect of EPL.

Note, however, that an unemployment rate of 24.8% is quite high and therefore the situation described, where the overall effect of EPL turns from positive to negative, pertains to certain regions. Not surprisingly, we find that the negative EPL implications triggered by an unemployment rate > 24.8% pertain to regions from Greece and Spain. More specifically, this situation is observed in 9 (Anatoliki Makedonia & Thraki, Attiki, Dytiki Ellada, Dytiki Makedonia, Ipeiros, Kentriki Makedonia, Kriti, Sterea Ellada, and Thessalia) of the 13 Greek regions and in 6 (Andalucía, Canarias, Castilla-la Mancha, Comunitat Valenciana, Extremadura, and Región de Murcia) of the 17 Spanish regions of the sample. These are regions from countries that, following 2010, undertook reforms to lessen their excessive employment protection. The lessening of employment protection, however, also led to an inevitable sharp increase in unemployment (see Pissarides 2013). Our findings thus suggest a mechanism where the subsequent sharp increase in unemployment affected (and reversed) the individual push effect of EPL on self-employment.

One could (rightfully) argue that the results may differ when detangling self-employment with employees (employers) and own account self-employment (own account workers). As such, we additionally estimated the models for employers and own account workers separately. The same core results were, however, obtained: the negative effect of unemployment, the positive effect of the EPL, and the negative effect of the cross-level interaction UNEMP*EPL were confirmed, for both types of self-employment rates. The only differences observed pertain to the significance of some control variables or a switch in the sign of a few of them. For example, the female share turns from negative to positive (but statistically insignificant) when it comes to own account workers (in the models including the country variables and the cross-level interaction) but remains negative when it comes to employers (in all specifications). Our core results are thus consistent when distinguishing between employers and own account workers.^8^ Total self-employment, however, seems to better grasp the more general implications during the Great Recession. On average, the employers’ rate was already declining from 2008 and continued to do so throughout the crisis. On the other hand, while the own account workers’ rate experienced some moderate (compared to that of the employers) decline between 2008 and 2009 (with a subsequent rise between 2010 and 2012), the major downward pressure in the relevant rate occurred from 2013 onward. In turn, those considerations indicate unfavorable conditions when it comes to employers, and increased exit rates from own account work after 2013. The latter may have been caused by two reasons. First, in countries and regions where the recovery started by the end of 2012 or 2013, individuals had the opportunity to exit and/or avoid self-employment. On the other hand, in countries and regions where the recession continued and even ended in a sovereign debt crisis (e.g., in Greece), exit from own account work could have been caused by the distortion in product and labor markets.

### A comment on the effect of the control variables

To begin with, the opportunity cost of self-employment is eminently evident in all relevant control variables. More specifically, in Table 3 GDP p.c. is negative in all specifications.^9^ According to Torrini (2005), a decline in returns to entrepreneurship relative to wages can be expected when economies become more capital intensive. Another explanation regarding the negative sign of GDP p.c. is provided by Chowdhury et al. (2015) who explain that, under the incidence of improved living standards, individuals may choose to maintain those improved standards, whereas those who did not experience an amelioration of their living standards may search for stable opportunities that will, in turn, discourage the risky transition to self-employment. In a similar vein, Bergmann and Sternberg (2007) argue that it may be the case that increased GDP per capita signals the existence of employment opportunities, thus making entrepreneurship less attractive. Therefore, our results indicate that regional self-employment would not be considered under better wage prospects. The expected return from entrepreneurship must thus become more attractive now, given that the opportunity cost of entrepreneurship itself has increased (e.g., see Amit et al. 1995; Foti and Vivarelli 1994; Knight 1921; Oxenfeldt 1943).^10^ Moreover, also notice that all coefficients regarding the employment share in all five industries we controlled for, indicate that on average paid employment would be preferred under the proper aggregate conditions (columns 2 to 4 of Table 3). Overall, both GDP p.c. and the employment in the five sectors we have included in the analysis, imply that during a severe recession better employment and wage prospects would disincentivize self-employment.

In columns 2 to 4 of Table 3, HC is positive and statistically significant, confirming the positive relationship usually found between an educated labor force and entrepreneurship (see Barreneche García 2014). In addition, we also confirm the negative relationship between self-employment and females (FMS) (see Georgellis and Wall 2000). Finally, population density (PDEN) is positive and statistically significant, indicating that opportunities arising from the local market size would encourage self-employment.

Moving on to the effect of the country-level variables, and as seen in columns 3 and 4 of Table 3, MW is negatively associated with self-employment. The negative sign of the MW indicates that the relevant legislation acts as a safety net since it guarantees standard minimum level of earnings obtained from paid employment. Bearing in mind that self-employment cannot guarantee any safety net regarding earnings, the minimum wage becomes an important opportunity cost. Moreover, and as already noted, the minimum wage can be an important labor cost (e.g., Coomes et al. 2013; Kwapisz 2019).

As regards the effect of the country-level dummy variables controlling for the environment of wage bargaining, the results are as expected. When social partners tend to coordinate and balance their wage-related claims, regional self-employment increases. In Table 3, COORD is positive and statistically significant in all models. This result is attributed to the possibility of settling on a market clearing wage level which, in turn, does not hurt self-employment through an increase in labor costs.

On the contrary, the dummy controlling for the increased government intervention in wage bargaining that is beyond simple consultation and conflict resolution (GOVINT), is negative and statistically significant in all columns of Table 3. This is a strong indication that increased government intervention in wage bargaining could push for wage increases, possibly due to ideological, political, and electoral incentives, thus (un)intentionally increasing the opportunity cost of self-employment. Hence, in labor markets where the wage level comes at the expense of entrepreneurship, entrepreneurial activity should be expected to decrease (see Dilli 2019).

OCT is also negative and statistically significant when the country-level variables are included (column 3 of Table 3), and statistically insignificant but still negative in the model including the cross-level interaction UNEMP*EPL (column 4 of Table 3). As we mentioned before, its effect can be seen as bidirectional. On the one hand, employers have flexible channels of adjustment to shocks (e.g., see Brändle and Heinbach 2013), which favors self-employment, while on the other, employees have more chances of maintaining their jobs, thus favoring paid employment. Despite the weak statistical significance, our results suggest that the latter effect prevails. This being the case, the flexibility in readjusting labor contracts can help workers maintain their jobs and avoid self-employment under turbulent times. In other words, any agreement between employers and employees that would have been perceived by the latter part mostly as a win–win game, would have barred transitions to regional self-employment. Finally, DCR, being positive (columns 3 and 4 of Table 3), confirms the suggested importance of access to capital for entrepreneurship (see Naudé et al. 2008).

### Robustness tests

In order to assess the robustness of our results, we proceed to the estimation of Eqs. 1 and 2 with three alternative samples. First, and drawing on the Varieties-of-Capitalism (VoC) literature, we drop UK, which is the only Liberal Market Economy (LME) in our sample. LMEs present highly deregulated labor markets with flexible labor market institutions, both with respect to employment protection and wage bargaining (see Dilli et al. 2018). Thus, leaving the sample only with regions and countries with more regulated labor markets than LMEs could potentially affect the results. Ideally, it would be interesting to reconduct the analysis by estimating more alternative samples: one without Coordinated Market Economies (CMEs), one without Mediterranean Market Economies (MMEs), and finally one without Eastern European Market Economies (EMEs). However, that would lead us to a decrease in the sample by seven (i.e., Austria, Belgium, Denmark, Finland, Germany, the Netherlands, and Sweden), five (i.e., France, Greece, Italy, Portugal, and Spain), and four (i.e., Czech Republic, Hungary, Poland, and Slovakia) countries, respectively, thus diminishing the reliability of the estimates due to the sample demands of the multilevel analysis itself (see Bryan and Jenkins 2016; Hox et al. 2017, pp. 212–218; McNeish and Wentzel 2017). As regards the results of the sample without UK, they have the exact same implications.

For the second alternative sample, and while also utilizing the information available by Eurofound (2017) for the countries included in our analysis, we dropped Netherlands and UK, that is, two countries that experienced an increase in self-employment during 2008–2015. On the contrary, for the last alternative sample, we dropped Portugal and Hungary, where the strongest decrease in self-employment was observed during the same period (with respect to the countries covered by Eurofound (2017) and also included in our analysis). As regards the sample without Netherlands and UK, the changes to mention are that in the models including all three levels and the cross-level interaction (UNEMP*EPL) OCT turn statistically insignificant (but still negative). When considering the sample without Portugal and Hungary, in the model including the cross-level interaction (UNEMP*EPL) PDEN (still positive) and OCT (still negative) turn statistically insignificant. The changes related to the statistical significance of the predictors could be driven purely by the reduction of the sample size, which is important for accurate estimations in multilevel analysis. Thus, we conclude that overall, the core implications of our results are robust to the changes made.

## Conclusion

The present research effort has contributed to the relevant literature by assessing the role of employment protection in affecting the relationship between regional self-employment and unemployment under conditions of economic hardship. More specifically, we argue that particularly during turbulent times, the effect of important determinants of regional self-employment, such as the regional unemployment rate, cannot be examined in isolation from parameters applicable nationwide. Although we controlled for a number of variables both at the regional and national level, we turned our attention to regional unemployment and we employed a meaningful interaction with the Employment Protection Legislation index.

From a methodological point of view, we argue that the inclusion of the country-level variables (i.e., the highest level in our analysis) and the cross-level interaction between regional unemployment and employment protection, provides useful insight and additional implications. Namely, we have shown that while the direct effect of regional unemployment already supports ‘prosperity-pull’ implications, its interaction with the Employment Protection Legislation index further magnifies the effect. In this sense we find evidence for the adverse combined effect of a recessionary period and the regulatory environment that promotes strict employment protection. It may be for that reason, that during the recent recession, many governments attempted to combat the negative shock by promoting flexible labor market reforms, even if it raised the question whether this was made at the expense of job security (see Heyes 2013). In addition, we have also shown that the push effect of strict employment protection is reversed once a specific threshold of unemployment has been attained. However, only regions (i.e., some Greek and Spanish) with extreme levels of unemployment show this outcome.

A broader conclusion drawn from the results pertaining to the variables we controlled for, is that during the recent recession regional self-employment would have been avoided under better employment and wage prospects. This is apparent in all variables capturing or approximating the opportunity cost of entrepreneurship. This is shown, for example, in the GDP per capita, the minimum wage, and the employment in any of the five sectors we controlled for. In this respect, it is critical to understand that the transition to self-employment is not the same as that to paid employment. The former requires an additional number of important prerequisites, including the available capital or the condition of the market that the prospective self-employed will operate. In the context of a severe recession, the situation can become even more hostile for self-employment, especially under the presence of increased labor costs and higher employment protection. On the contrary, it would be much easier to pursue self-employment under a cooperative environment between employers and employees, and greater access to capital.

Of course, further research is needed upon the availability of relevant data that will be able to support a multilevel analysis of this kind. For instance, different types of entrepreneurship should be examined. Finally, although this was a first effort to shed light on the implications of employment protection on the occupational choice of regional self-employment during the Great Recession, the effect on certain regions, within and across countries, would be also fruitful to examine.

### Electronic supplementary material

Below is the link to the electronic supplementary material.

Supplementary file1 (DOCX 30 kb)

## Appendix

See Tables 4  and 5 .

**Table 4:** Correlation matrix

Variables	(1)	(2)	(3)	(4)	(5)	(6)	(7)	(8)	(9)	(10)	(11)	(12)	(13)	**(14)**	**(15)**	**(16)**	**(17)**
(1) SER	1.00																
(2) UNEMP	0.31	1.00															
(3) GDP p.c	− 0.29	− 0.42	1.00														
(4) IND	− 0.17	− 0.22	− 0.22	1.00													
(5) CON	0.20	0.01	− 0.46	0.13	1.00												
(6) WRTAC	0.27	0.22	0.19	− 0.51	− 0.02	1.00											
(7) FBS	− 0.35	− 0.32	0.74	− 0.46	− 0.45	0.19	1.00										
(8) NMS	− 0.41	0.01	0.16	− 0.51	− 0.20	− 0.23	0.32	1.00									
(9) HC	− 0.32	− 0.12	0.50	− 0.42	− 0.33	0.15	0.61	0.36	1.00								
(10) PDEN	− 0.10	0.00	0.42	− 0.26	− 0.35	0.14	0.51	0.13	0.26	1.00							
(11) FSH	− 0.57	− 0.33	0.35	− 0.13	− 0.29	− 0.06	0.39	0.31	0.54	0.09	1.00						
(12) EPL	0.33	0.36	− 0.08	0.09	0.10	− 0.14	− 0.25	− 0.08	− 0.42	− 0.13	− 0.34	1.00					
(13) MW	0.15	0.25	− 0.33	− 0.15	0.12	0.13	− 0.02	− 0.15	0.18	0.02	0.06	− 0.04	1.00				
(14) COORD	− 0.03	− 0.07	0.43	− 0.05	− 0.22	− 0.01	0.17	0.20	0.01	0.05	− 0.15	0.24	− 0.65	1.00			
(15) GOVINT	0.32	0.27	− 0.09	− 0.20	0.02	− 0.11	− 0.03	0.22	− 0.18	− 0.05	− 0.20	0.58	0.03	− 0.01	1.00		
(16) OCT	− 0.04	− 0.18	0.10	0.26	− 0.12	− 0.19	− 0.03	− 0.10	− 0.20	0.01	− 0.14	0.09	− 0.47	0.40	− 0.14	1.00	
(17) DCR	− 0.07	0.09	0.06	− 0.35	0.05	0.31	0.12	0.24	0.37	− 0.02	0.11	− 0.45	0.06	− 0.07	− 0.27	− 0.26	1.00

**Table 5:** List of EU countries (17) and regions (230) used in the analysis. NUTS-2 classification

Countries	Regions
Austria	AT11—Burgenland (AT), AT12—Niederösterreich, AT13—Wien, AT21—Kärnten, AT22—Steiermark, AT31—Oberösterreich, AT32—Salzburg, AT33—Tirol, AT34—Vorarlberg
Belgium	BE10—Région de Bruxelles-Capitale/Brussels Hoofdstedelijk Gewest, BE21—Prov. Antwerpen, BE22—Prov. Limburg (BE), BE23—Prov. Oost-Vlaanderen, BE24—Prov. Vlaams-Brabant, BE25—Prov. West-Vlaanderen, BE31—Prov. Brabant wallon, BE32—Prov. Hainaut, BE33—Prov. Liège, BE34—Prov. Luxembourg (BE), BE35—Prov. Namur
Czech Republic	CZ01—Praha, CZ02—Strední Cechy, CZ03—Jihozápad, CZ04—Severozápad, CZ05—Severovýchod, CZ06—Jihovýchod, CZ07—Strední Morava, CZ08—Moravskoslezsko
Denmark	DK01—Hovedstaden, DK02—Sjælland, DK03—Syddanmark, DK04—Midtjylland, DK05—Nordjylland
Finland	FI19—Länsi-Suomi, FI1B—Helsinki-Uusimaa, FI1C—Etelä-Suomi, FI1D—Pohjois- ja Itä-Suomi
France	FR10—Île de France, FRB0—Centre—Val de Loire, FRC1—Bourgogne, FRC2—Franche-Comté, FRD1—Basse-Normandie, FRD2—Haute-Normandie, FRE1—Nord-Pas-de-Calais, FRE2—Picardie, FRF1—Alsace, FRF2—Champagne-Ardenne, FRF3—Lorraine, FRG0—Pays-de-la-Loire, FRH0—Bretagne, FRI1—Aquitaine, FRI2—Limousin, FRI3—Poitou–Charentes, FRJ1—Languedoc-Roussillon, FRJ2—Midi-Pyrénées, FRK1—Auvergne, FRK2—Rhône-Alpes, FRL0—Provence-Alpes-Côte d'Azur
Germany	DE11—Stuttgart, DE12—Karlsruhe, DE13—Freiburg, DE14—Tübingen, DE21—Oberbayern, DE22—Niederbayern, DE23—Oberpfalz, DE24—Oberfranken, DE25—Mittelfranken, DE26—Unterfranken, DE27—Schwaben, DE30—Berlin, DE40—Brandenburg, DE50 – Bremen, DE60—Hamburg, DE71—Darmstadt, DE72—Gießen, DE73—Kassel, DE80—Mecklenburg-Vorpommern, DE91—Braunschweig, DE92—Hannover, DE93—Lüneburg, DE94—Weser-Ems, DEA1—Düsseldorf, DEA2—Köln, DEA3—Münster, DEA4—Detmold, DEA5—Arnsberg, DEB1—Koblenz, DEB2—Trier, DEB3—Rheinhessen-Pfalz, DEC0—Saarland, DED2—Dresden, DED4—Chemnitz, DED5—Leipzig, DEE0—Sachsen-Anhalt, DEF0—Schleswig–Holstein, DEG0—Thüringen
Greece	EL30—Attiki, EL41—Voreio Aigaio, EL42—Notio Aigaio, EL43—Kriti, EL51—Anatoliki Makedonia, Thraki, EL52—Kentriki Makedonia, EL53—Dytiki Makedonia, EL54—Ipeiros, EL61—Thessalia, EL62—Ionia Nisia, EL63—Dytiki Ellada, EL64—Sterea Ellada, EL65—Peloponnisos
Hungary	HU21—Közép-Dunántúl, HU22—Nyugat-Dunántúl, HU23—Dél-Dunántúl, HU31—Észak-Magyarország, HU32—Észak-Alföld, HU33—Dél-Alföld
Italy	ITC1—Piemonte, ITC2—Valle d'Aosta/Vallée d'Aoste, ITC3—Liguria, ITC4—Lombardia, ITF1—Abruzzo, ITF2—Molise, ITF3—Campania, ITF4—Puglia, ITF5—Basilicata, ITF6—Calabria, ITG1—Sicilia, ITG2—Sardegna, ITH1—Provincia Autonoma di Bolzano/Bozen, ITH2—Provincia Autonoma di Trento, ITH3—Veneto, ITH4—Friuli-Venezia Giulia, ITH5—Emilia-Romagna, ITI1—Toscana, ITI2—Umbria, ITI3—Marche, ITI4—Lazio
Netherlands	NL11—Groningen, NL12—Friesland (NL), NL13—Drenthe, NL21—Overijssel, NL22—Gelderland, NL23—Flevoland, NL31—Utrecht, NL32—Noord-Holland, NL33—Zuid-Holland, NL34—Zeeland, NL41—Noord-Brabant, NL42—Limburg (NL)
Poland	PL21—Malopolskie, PL22—Slaskie, PL41—Wielkopolskie, PL42—Zachodniopomorskie, PL43—Lubuskie, PL51—Dolnoslaskie, PL52—Opolskie, PL61—Kujawsko-Pomorskie, PL62—Warminsko-Mazurskie, PL63—Pomorskie, PL71—Lódzkie, PL72—Swietokrzyskie, PL81—Lubelskie, PL82—Podkarpackie, PL84—Podlaskie
Portugal	PT11—Norte, PT15—Algarve, PT16—Centro (PT), PT17—Área Metropolitana de Lisboa, PT18—Alentejo
Slovakia	SK01—Bratislavský kraj, SK02—Západné Slovensko, SK03—Stredné Slovensko, SK04—Východné Slovensko
Spain	ES11—Galicia, ES12—Principado de Asturias, ES13—Cantabria, ES21—País Vasco, ES22—Comunidad Foral de Navarra, ES23—La Rioja, ES24—Aragón, ES30—Comunidad de Madrid, ES41—Castilla y León, ES42—Castilla-la Mancha, ES43—Extremadura, ES51—Cataluña, ES52—Comunitat Valenciana, ES53—Illes Balears, ES61—Andalucía, ES62—Región de Murcia, ES70—Canarias
Sweden	SE11—Stockholm, SE12—Östra Mellansverige, SE21—Småland med öarna, SE22—Sydsverige, SE23—Västsverige, SE31—Norra Mellansverige, SE32—Mellersta Norrland, SE33—Övre Norrland
UK	UKC1—Tees Valley and Durham, UKC2—Northumberland and Tyne and Wear, UKD1—Cumbria, UKD3—Greater Manchester, UKD4—Lancashire, UKD6—Cheshire, UKD7—Merseyside, UKE1—East Yorkshire and Northern Lincolnshire, UKE2—North Yorkshire, UKE3—South Yorkshire, UKE4—West Yorkshire, UKF1—Derbyshire and Nottinghamshire, UKF2—Leicestershire, Rutland and Northamptonshire, UKF3—Lincolnshire, UKG1—Herefordshire, Worcestershire and Warwickshire, UKG2—Shropshire and Staffordshire, UKG3—West Midlands, UKH1—East Anglia, UKH2—Bedfordshire and Hertfordshire, UKH3—Essex, UKJ1—Berkshire, Buckinghamshire and Oxfordshire, UKJ2—Surrey, East and West Sussex, UKJ3—Hampshire and Isle of Wight, UKJ4—Kent, UKK1—Gloucestershire, Wiltshire and Bristol/Bath area, UKK2—Dorset and Somerset, UKK3—Cornwall and Isles of Scilly, UKK4—Devon, UKL1—West Wales and The Valleys, UKL2—East Wales, UKM5—North Eastern Scotland, UKM6—Highlands and Islands, UKN0—Northern Ireland (UK)

## Footnotes

1 According to the OECD “The OECD indicators of employment protection legislation evaluate the regulations on the dismissal of workers on regular contracts and the hiring of workers on temporary contracts. They cover both individual and collective dismissals.”

2 However, increased coordination among social partners does not rule out the possibility of a higher degree of involvement of a certain part, usually that of the government, which renders necessary the presence of both COORD and GOVINT. The reasoning is that even under an environment of increased coordination, there is a significant chance that minor players tend to follow the decisions of major players (Kenworthy 2001), which could lead to a binding wage agreement, but it is ambiguous whether it would involve fewer disputes and greater compliance in the labor market.

3 To combat multicollinearity problems, we stick only to the categorization concerning the coordination in wage setting. Moreover, the fact that firms within the same industry/region/country may endorse different types of VoC or display diverse levels of innovativeness (e.g., see Herrmann 2019; Schneider and Paunescu 2012), makes it difficult to introduce the VoC argument into our research. This would demand a more focused investigation that is, however, well beyond the intentions of our effort.

4 An option could be (e.g., for checking the robustness of the results) to estimate a fixed effects model (FEM). One major limitation of the FEM, however, is that although we could conduct an analysis to check the robustness of potential cross-level interactions, we would not be able to measure the direct effect of the country-level predictors, since the country intercepts already account for cross-country differences. For this reason, in this study we did not consider a FEM model.

5 Notice that we have also estimated more dynamic versions of Eqs. 1 and 2, which however did not yield meaningful results (i.e., mainly in terms of statistical significance). As a matter of fact, we first conducted ANOVA and MANOVA, which both showed that time is not a significant source of systematic variation.

6 Note here that we have additionally investigated whether the effect of UNEMP is non-linear. As such, we have used the square of regional unemployment as in Georgellis and Wall (2000), but still obtain a negative sign, both in UNEMP and squared UNEMP. In view of this, no evidence for non-linear effects of UNEMP was found. One possible explanation for this finding is the already high unemployment rate during the majority of the years of the sample period.

7 The possibility of the existence of a non-linear relationship between EPL and regional self-employment was examined too. However, and by taking the square of EPL, we still obtain a positive sign, confirming the individual push effect of strict EPL during a recessionary period.

8 See our online Supplementary Material appendix for the relevant results.

9 As in the case of UNEMP and EPL, we examined whether a potential U-shaped relationship exists between regional GDP p.c. and self-employment. While positive, no significant effect of the square of GDP p.c. was found.

10 As regards regional incomes, we have estimated all estimations by replacing regional GDP per capita with regional compensation of employees (European Commission, ARDECO). The results and implications are the same. We do not include both predictors in the same model since together they increase VIF way above 10 (see Kutner et al. 2004). Thus, regional compensation of employees, which accounts for the regional wage bill, confirms that better employment and wage prospects would disincentivize self-employment.
