# Supplementary material for: Employment protection and regional self-employment rates in an economic downturn: a multilevel analysis
Source: Ann Reg Sci. 2023 Feb 25:1–30. Online ahead of print. doi: 10.1007/s00168-023-01214-5 (PMC9959941; doi:10.1007/s00168-023-01214-5)
Supplement: Supplementary file 1 — Supplementary file1 (DOCX 30 kb) [file 168_2023_1214_MOESM1_ESM.docx]

**Supplementary Material**

Here we briefly present supplementary results pertaining to own account workers and employers. Starting with own account workers, and as seen in Table S1, the core results are identical (in terms of implications and interpretation) to those obtained from the main analysis (i.e., for total self-employment). That is, UNEMP is negative, EPL is positive, and the cross-level interaction is negative. In terms of the unemployment rate that would turn the positive effect of EPL to negative, our results show that an unemployment rate of >17.6% would be needed for such a change. Recall that for the main results (i.e., for total self-employment), the relevant turning point is 24.8%. Finally, and as regards the control variables, the majority of these match the main results. The three exceptions are the female share (FSH) (i.e., sign change, see Columns 3 and 4 of Table S1; loss of statistical significance, see Columns 2, 3 and 4 of Table S1), the incidence of increased government intervention in wage bargaining that is beyond simple consultation and conflict resolution (GOVINT) (loss of statistical significance, see Column 4 of Table S1), and the DCR, the domestic credit provided by banks to the private sector (loss of statistical significance, see Columns 3 and 4 of Table S1).

In respect of the results pertaining to employers (Table S2), we again see that the core results are identical (in terms of implications and interpretation) to those obtained for total self-employment (UNEMP is negative, EPL is positive, and the cross-level interaction is negative). In the case of employers, the unemployment rate that would alter the push effect of EPL is a rate of >36.7%, which is, however, an unemployment rate not observed in the sample. Regarding the control variables, and as seen in Table S2, in many cases the results diverge from the main analysis (i.e., in the sign and/or the statistical significance). Yet, our core results remain consistent in any case regardless of the dependent variable, thus confirming the reasoning behind the investigation of the relationship between self-employment, unemployment and employment protection.

| Table S1. Labor market determinants of regional Own account workers’ rate (OWR). Multilevel model (MLM) estimates | | | | |
| --- | --- | --- | --- | --- |
|  | **(1)** | **(2)** | **(3)** | **(4)** |
| **Regional-level variables** | | | | |
| **UNEMP** |  | -0.09039***  (0.01052) | -0.06210***  (0.01221) | -0.05216***  (0.01282) |
| GDP p.c. |  | -0.00048***  (0.00013) | -0.00045***  (0.00014) | -0.00045***  (0.00014) |
| IND |  | -0.51013***  (0.02675) | -0.50747***  (0.02692) | -0.50999***  (0.02696) |
| CON |  | -0.37895***  (0.03746) | -0.38179***  (0.04123) | -0.38520***  (0.04121) |
| WRTAC |  | -0.44404***  (0.02801) | -0.45243***  (0.02822) | -0.45450***  (0.02823) |
| FBS |  | -0.39195***  (0.03377) | -0.37774***  (0.03415) | -0.36916***  (0.03431) |
| NMS |  | -0.51435***  (0.02935) | -0.52354***  (0.02975) | -0.52781***  (0.02980) |
| HC |  | 0.09178***  (0.00951) | 0.09121***  (0.00998) | 0.09015***  (0.00997) |
| PDEN |  | 0.00001***  (0.00000) | 0.00001***  (0.00000) | 0.00000***  (0.00000) |
| FSH |  | -0.00078  (0.02970) | 0.01609  (0.02972) | 0.02054  (0.02974) |
|  |  |  |  |  |
| **Country-level variables** | | | | |
| **EPL** |  |  | 0.00396**  (0.00162) | 0.00392**  (0.00162) |
| MW |  |  | -0.00340**  (0.00135) | -0.00338**  (0.00135) |
| COORD |  |  | 0.00472***  (0.00132) | 0.00424***  (0.00133) |
| GOVINT |  |  | -0.00210**  (0.00102) | -0.00160  (0.00104) |
| OCT |  |  | -0.00102*  (0.00062) | -0.00093  (0.00062) |
| DCR |  |  | 0.00115  (0.00175) | 0.00073  (0.00175) |
|  |  |  |  |  |
| **Cross-level interaction** | | | | |
| **UNEMP*EPL** |  |  |  | -0.02228**  (0.00893) |
|  |  |  |  |  |
| Constant | 0.09407***  (0.01109) | 0.09196***  (0.00927) | 0.09144***  (0.00914) | 0.09187***  (0.00914) |
| Country level variance | 0.00204  (0.00074) | 0.00144  (0.00052) | 0.00140  (0.00051) | 0.00140  (0.00051) |
| Regional level variance | 0.00046  (0.00005) | 0.00018  (0.00002) | 0.00018  (0.00002) | 0.00019  (0.00002) |
| Residual variance | 0.00006  (2.18E-06) | 0.00005  (1.88E-06) | 0.00005  (1.85E-06) | 0.00005  (1.84E-06) |
| Average VIF |  | 2.41 | 2.84 | 3.00 |
| N | 1840 | 1840 | 1840 | 1840 |
| Countries \| Regions | 17 \| 230 | 17 \| 230 | 17 \| 230 | 17 \| 230 |
| ICC | 0.79686 | 0.85954 | 0.85508 | 0.85492 |
| Log restricted-likelihood | 5795.6665 | 5979.2904 | 5961.1953 | 5960.5 |
| Wald χ^2^ test |  | 599.46*** | 634.35*** | 640.66*** |
| LR test vs. linear regression | 5774.47*** | 4151.99*** | 3925.13*** | 3914.04*** |
| Standard errors are in parentheses; *** p<.01, ** p<.05, * p<.1; a= For presentation purposes PDEN was rescaled to stand for 1000 persons/km^2^, while GDP p.c. was multiplied by 1000. | | | | |

| Table S2. Labor market determinants of regional Employers’ rate (EMPR). Multilevel model (MLM) estimates | | | | |
| --- | --- | --- | --- | --- |
|  | **(1)** | **(2)** | **(3)** | **(4)** |
| **Regional-level variables** | | | | |
| **UNEMP** |  | -0.12198***  (0.00590) | -0.09247***  (0.00659) | -0.08647***  (0.00702) |
| GDP p.c. |  | -0.00018***  (0.00007) | -0.00017**  (0.00007) | -0.00016**  (0.00007) |
| IND |  | -0.01139  (0.01119) | -0.01159  (0.01127) | -0.01384  (0.01129) |
| CON |  | 0.00492  (0.01938) | -0.00721  (0.02090) | -0.00937  (0.02088) |
| WRTAC |  | 0.06632***  (0.01236) | 0.06126***  (0.01245) | 0.05999***  (0.01245) |
| FBS |  | -0.05174***  (0.01513) | -0.04788***  (0.01515) | -0.04615***  (0.01515) |
| NMS |  | 0.00785  (0.01312) | -0.00477  (0.01316) | -0.00750  (0.01319) |
| HC |  | -0.00330  (0.00524) | 0.00021  (0.00533) | -0.00009  (0.00532) |
| PDEN |  | 0.00000  (0.00000) | -0.00000  (0.00000) | -0.00000  (0.00000) |
| FSH |  | -0.07197***  (0.01575) | -0.05731***  (0.01562) | -0.05749***  (0.01560) |
|  |  |  |  |  |
| **Country-level variables** | | | | |
| **EPL** |  |  | 0.00456***  (0.00088) | 0.00451***  (0.00087) |
| MW |  |  | -0.00195**  (0.00078) | -0.00193**  (0.00077) |
| COORD |  |  | 0.00650***  (0.00077) | 0.00626***  (0.00077) |
| GOVINT |  |  | -0.00120**  (0.00060) | -0.00092  (0.00061) |
| OCT |  |  | -0.00002  (0.00036) | 0.00003  (0.00036) |
| DCR |  |  | 0.00248***  (0.00093) | 0.00224**  (0.00093) |
|  |  |  |  |  |
| **Cross-level interaction** | | | | |
| **UNEMP*EPL** |  |  |  | -0.01230**  (0.00506) |
|  |  |  |  |  |
| Constant | 0.04042***  (0.00257) | 0.04070***  (0.00276) | 0.04025***  (0.00204) | 0.04048***  (0.00201) |
| Country level variance | 0.00011  (0.00004) | 0.00013  (0.00005) | 0.00007  (0.00003) | 0.00007  (0.00003) |
| Regional level variance | 0.00004  (3.88E-06) | 0.00002  (2.60E-06) | 0.00002  (2.73E-06) | 0.00002  (2.72E-06) |
| Residual variance | 0.00003  (1.10E-06) | 0.00002  (7.01E-07) | 0.00002  (6.55E-07) | 0.00002  (6.54E-07) |
| Average VIF |  | 2.41 | 2.84 | 3.00 |
| N | 1840 | 1840 | 1840 | 1840 |
| Countries \| Regions | 17 \| 230 | 17 \| 230 | 17 \| 230 | 17 \| 230 |
| ICC | 0.61587 | 0.74962 | 0.61558 | 0.60755 |
| Log restricted-likelihood | 6633.7719 | 7006.1676 | 7026.7804 | 7025.3585 |
| Wald χ^2^ test |  | 1078.30*** | 1264.23*** | 1273.57*** |
| LR test vs. linear regression | 2713.70*** | 2419.24*** | 1369.71*** | 1375.33*** |
| Standard errors are in parentheses; *** p<.01, ** p<.05, * p<.1; a= For presentation purposes PDEN was rescaled to stand for 1000 persons/km^2^, while GDP p.c. was multiplied by 1000. | | | | |
